# Nanodrugs Manipulating Endoplasmic Reticulum Stress for Highly Effective Antitumor Therapy

**DOI:** 10.3389/fphar.2022.949001

**Published:** 2022-07-12

**Authors:** Yuting Xiang, Min Liu, Yunrong Yang, Yubo Wang, Yige Qiu, Shiqi Tu, Yitian Jiang, Yayun Nan, Xiaojie Zhang, Qiong Huang

**Affiliations:** ^1^ Xiangya School of Pharmaceutical Sciences, Central South University, Changsha, China; ^2^ Hunan Provincial Key Laboratory of Cardiovascular Research, Xiangya School of Pharmaceutical Sciences, Central South University, Changsha, China; ^3^ Department of Pharmacy, Xiangya Hospital, Central South University, Changsha, China; ^4^ National Clinical Research Center for Geriatric Disorders, Xiangya Hospital, Central South University, Changsha, China; ^5^ Geriatric Medical Center, People’s Hospital of Ningxia Hui Autonomous Region, Yinchuan, China

**Keywords:** endoplasmic reticulum stress, tumor, immunogenic cell death, nanodrugs, photodynamic therapy

## Abstract

Cancer is one of the leading causes of death worldwide due to high morbidity and mortality. Many attempts and efforts have been devoted to fighting cancer. Owing to the significant role of the endoplasmic reticulum (ER) in cell function, inducing ER stress can be promising for cancer treatment. However, the sustained activation of cytoprotective unfolded protein response (UPR) presents a tremendous obstacle for drugs in inducing unsolved ER stress in tumor cells, especially small-molecule drugs with poor bioavailability. Therefore, many emerging nanodrugs inducing and amplifying ER stress have been developed for efficient cancer treatment. More importantly, the novel discovery of ER stress in immunogenic cell death (ICD) makes it possible to repurpose antitumor drugs for immunotherapy through nanodrug-based strategies amplifying ER stress. Therefore, this mini-review aims to provide a comprehensive summary of the latest developments of the strategies underlying nanodrugs in the treatment of cancer *via* manipulating ER stress. Meanwhile, the prospects of ER stress–inducing nanodrugs for cancer treatment are systematically discussed, which provide a sound platform for novel therapeutic insights and inspiration for the design of nanodrugs in treating cancer.

## Introduction

Consistently, cancer treatment is a long-standing conundrum within the field of medicine. Currently, the main treatment modalities for cancer are still based on chemotherapy, radiotherapy, and surgery. However, these traditional treatments elicit significant side effects and even tissue damage. For example, chemotherapy often causes severe hepatotoxicity and nephrotoxicity (Zhao et al., 2018; Zhao et al., 2022a; Liu et al., 2022; Xiao et al., 2022), which are responsible for severe deleterious hepatic and renal dysfunctions in patients. Therapeutic strategies targeting key organelles have attracted considerable attention, on account of the important role of organelles in maintaining the normal physiological function of cells. The endoplasmic reticulum (ER) is a central organelle that carries out many important functions such as synthesizing and folding proteins, modifying secreted and transmembrane proteins, regulating lipid synthesis and metabolism, storing calcium, and mediating signal transduction (King and Wilson, 2020). In addition, ER is closely apposed and dynamically tethered to other organelles through membrane networks, such as the nucleus, mitochondria, and Golgi apparatus (Chen and Cubillos-Ruiz, 2021). Once the ER is injured, the physiology of the entire cell gets adversely affected. Therefore, the tightly regulated process of ER function is crucial for cell fate determination.

Many factors, such as intracellular reactive oxygen species (ROS) and nutrient deprivation, disturb ER functioning in inducing ER stress based on the accumulation of unfolded and misfolded proteins in the ER (Yao et al., 2017). The initial adaptive mechanisms such as unfolded protein response (UPR) in tumor cells provide a possibility to restore and maintain protein homeostasis, but unsolved ER stress still leads to cell death (Chen and Cubillos-Ruiz, 2021). Some clinical chemotherapeutics are found to be associated with ER stress induction, while the upregulation of UPR promotes resistance (Bahar et al., 2019). Therefore, many drugs have been proposed to induce and exacerbate severe ER stress in killing tumor cells as potential therapeutics. For example, molecular chaperone binding immunoglobulin (BiP), an ER stress sensor, is highly expressed in mediating tumor chemotherapy resistance by activating UPR to restore ER homeostasis in cancer cells (Hetz et al., 2020). Currently, many BiP inhibitors have been developed for killing tumors, such as KP1339 (Wernitznig et al., 2019) and HA15 (Cerezo et al., 2016).

However, the clinical application of these ER-targeted small-molecule drugs faces great bottlenecks, such as the lack of tumor targeting and strong side effects. The development of nanodrugs offers a possibility to address the dilemma in traditional drugs (Shi et al., 2021). The enhanced penetration and retention (EPR) effects mediate the accumulation of nanodrugs at tumor sites (Huang et al., 2022) to reduce the risk of toxicity in normal tissues. Moreover, nanodrugs can be modified with specific ligands targeting tumor cells or ER (Li et al., 2020) to further improve the enrichment of nanodrugs in tumor sites (Yang et al., 2022) and anticancer efficacy. Notably, nanodrugs can induce immunogenic cell death (ICD) to effectively improve immunotherapy by amplifying ER stress. Specifically, when the nanodrugs disrupt the ER, ER stress induces imbalances in calcium homeostasis, and calreticulin (CRT) transfers from the ER to the cell membrane to act as an “eat-me” signal, inducing inflammatory cell infiltration and enhancing tumor cell antigens presented.

**FIGURE 1:**
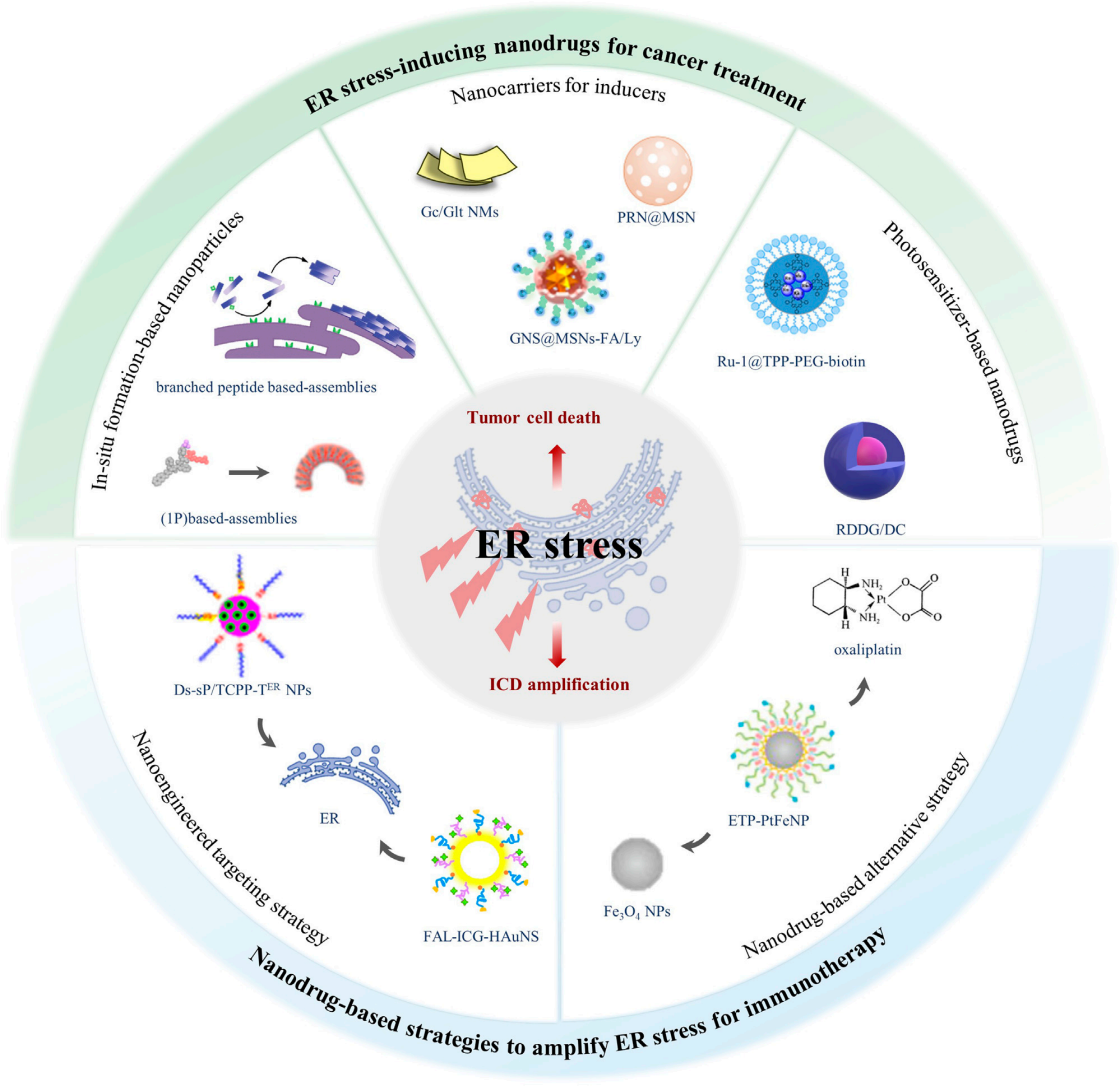
The scope and focus of this article. Accumulation of misfolded protein results in ER stress, which can induce antitumor effects by triggering tumor cell death and amplifying ICD. ER stress–inducing nanodrugs can treat cancer through activating unsolved ER stress. In addition, nanodrugs amplifying ER stress *via* ER-targeting strategy and combination strategy make it possible for traditional ICD inducers to initiate antitumor immunotherapy.

To our knowledge, no similar reviews have been published in summarizing newly developed nanodrugs based on ER stress for cancer treatment. Herein, this review covers the recent progress in oncology therapeutics about nanodrugs for ER stress induction, recapitulating the design of nanodrugs that induce ER stress, together with the strategies of nanodrugs that amplify ER stress to repurpose antitumor drugs for cancer immunotherapy (Figure 1) (Table 1). Finally, the obstacles and prospects of ER stress–based nanodrugs for cancer treatment are discussed.

**TABLE 1:** Nanodrugs manipulating ER stress for highly effective antitumor therapy.

Category	Nanodrugs	Factors that induce or amplify ER stress	Tumor/ER-targeting strategies	Ref.
**ER stress–inducing nanodrugs for cancer treatment**				
*In situ* formation–based ER-targeted nanoparticles	Phosphotetrapeptide (1P)-based assemblies	\	d-phosphotyrosine	Feng et al. (2018)
	branched peptide (1)–based assemblies	\	KYDKKKKDG substance	Kim et al. (2021)
	1-Nap nanofiber	\	RVRR substance	Fu et al. (2020)
Nanocarriers with small-molecule drugs	Cc/Glt NM	Curcumin	\	Cheng et al. (2020)
	PRN@MSN	Propranolol;	\	Wu et al. (2020)
		Mesoporous silica nanoparticle		
	GNS@MSNs-FA/Ly	Gold nanostars;	Folate	Hu et al. (2021)
		Lycorine		
Multifunctional photosensitizer–based nanodrugs	Ru-1@TPP-PEG-biotin SAN	Ruthenium complex 1;	Biotin	Purushothaman et al. (2020)
		Tetraphenylporphyrin		
	RDDG/DC	2-deoxy-glucose;	ROS-sensitive bond	Dong et al. (2019)
		Dithiophene-benzotriazole groups		
**Nanodrug-based strategies to amplify ER stress for immunotherapy**				
ER-targeting nanodrugs based on ICD inducers	Ds-sP/TCPP-TER NPs	Meso-tetra(4-carboxyphenyl) porphyrin	Reduction-sensitive polymeric;	Deng et al. (2020)
	FAL-ICG-HAuNS	Indocyanine green;	*p*-toluene sulfonyl group	Li et al. (2019)
		Gold nanospheres	Pardaxin peptides	
ER stress–inducing nanodrugs combined with ICD inducers	3-NPs	Cisplatin;	pH sensitivity;	Liu et al. (2019)
		Adjudin;	GSH sensitivity	
		WKYMVm		
	ETP-PtFeNP	Oxaliplatin;	α-enolase targeting peptide	Chen et al. (2019)
		Magnetic nanoparticle		

## Endoplasmic Reticulum Stress–Inducing Nanodrugs for Cancer Treatment

ER stress is a cellular condition characterized by the unsolved accumulation of misfolded proteins, which is detrimental to the organism. Misfolded proteins have a higher affinity to molecular chaperone BiP and activate sensors of ER stress *via* titrating BiP from sensors [PKR-like ER kinase (PERK), activating transcription factor 6 (ATF6), and inositol-requiring enzyme 1 (IRE1α)], termed UPR. Subsequently, the activation of sensors initiate cytoprotective mechanisms, including inducing transcription of the cell-protective molecule, directing the protein to the ubiquitin-proteasome system (UPS), promoting protective autophagy, and so on. Therefore, there are two strategies to induce ER stress as expected: promoting the production of misfolded proteins and inhibiting UPR-mediated protective effects (Marciniak et al., 2022). Currently, many emerging nanodrugs targeting ER have been developed for efficient cancer treatment. According to the pharmacological mechanism, these nanodrugs are mainly divided into the following three categories: the first category, the *in situ* formation-based ER-targeted nanoparticles to induce ER stress by enzyme-instructed self-assembly (EISA); the second category, nanocarriers for small-molecule drugs substantially induce ER stress by improving the targeting efficiency; the third category, photodynamic therapy (PDT)–based nanodrugs amplify ER stress by generating uncontrolled ROS.

### *In Situ* Formation–Based Endoplasmic Reticulum–Targeted Nanoparticles

EISA technology is an effective method for *in situ* self-assembly, which is a benefit for organelles targeting and inducing stress (Ji et al., 2021). The overexpressed enzymes in tumor cells can achieve targeted enrichment and improve selectivity for some molecules containing specific amino acid sequence substrates for these enzymes. These specially designed molecules can self-assemble into ER-targeted nanoforms to induce ER stress through membrane lipid action in cancer cells after the substrate fragments are cleaved by these enzymes (Gao et al., 2020; Xiang et al., 2022a). Recently, Feng et al. (2018) adopted alkaline phosphatase (ALP), highly expressed in tumor cells (HeLa cells), to construct an EISA-related phosphotetrapeptide (1P)-based ER inducer for cancer therapy. The 1P precursor contained a d-phosphotyrosine as a specific substrate of ALP, and positively charged l-homoarginine, which targeted the ER. After the 1P precursor was catalyzed by ALP to generate 1P, 1P self-assembled on the surface of the cancer cell membrane to form unique crescent-shaped 1P nanoparticles. Subsequently, the 1P nanoparticles were selectively enriched in the ER to induce ER stress. Similarly, Kim et al. (2021) utilized trypsin-1, which was overexpressed in the ER of a high-grade serous ovarian cancer cell line (OVSAHO), to develop a trypsin-1 (PRSS1)–based branched peptide chain. After proteolysis, the branched peptide chains formed peptide assemblies to accumulate in the ER, upregulated ER stress-related proteins, and killed OVSAHO cells through various death pathways. In addition, furin that is highly expressed in many malignant tumor cells (Fu et al., 2020) has been used to treat cancer. Based on its location in the trans-Golgi network, furin-instruct EISA blocked the transport of ER to the Golgi apparatus to induce ER stress and cancer cell death.

### Nanocarriers With Small-Molecule Drugs

Nanocarriers were also developed for ER-targeted small-molecule drugs to improve their efficacy in tumors (Wang et al., 2021). Cheng et al. (2020) encapsulated curcumin in gelatin-blended nanofibrous mat (Cc/Glt NM) to address the inherent insolubility, instability, poor absorption, and rapid systemic elimination of curcumin. Cc/Glt NM effectively entered cancer cells to release curcumin, which activated the BiP/p-PERK/p-elF2a pathway to induce ER stress. As expected, Cc/Glt NM significantly reduced the tumor volume in pancreatic adenocarcinoma (PDAC) tumor–bearing mice with enhanced ER stress levels after topical application. In addition, Wu et al. (2020) adopted mesoporous silica nanoparticle (MSN) to deliver propranolol (PRN), the first-line therapy for hemangiomas. PRN@MSN strongly inhibited the formation of microvessels in murine hemangioma models *via* increased ER stress, where MSN induced ER stress and PRN inhibited autophagy that was resistant to ER stress. Moreover, Hu et al. (2021) integrated gold nanostars (GNS) and the antineoplastic drug lycorine (Ly) into MSNs with modified tumor-targeted folate (FA). GNS@MSNs-FA/Ly exhibited highly specific tumor growth inhibition in osteosarcoma cell tumor–bearing mice without additional side effects, among which Ly promoted mitochondrial dysfunction to interfere with ER *via* endoplasmic reticulum–mitochondrial contact (Giacomello et al., 2020).

### Multifunctional Photosensitizer-Based Nanodrugs

PDT is one of the potential therapeutic strategies for cancers that generates cytotoxic ROS by the reaction of photosensitizers with oxygen (O_2_) under the light (Zhao et al., 2022b; Long et al., 2022). The ROS generated during PDT attack proteins to form toxic ROS-modified proteins, which can provoke ER stress by inducing the accumulation of toxic protein (Chen et al., 2021; Zhu et al., 2022) in the ER. In addition, excessive intracellular ROS affect ER-resident calcium channels (Görlach et al., 2015) and promote lipid peroxidation (Vladykovskaya et al., 2012), which also disturb the homeostasis of the ER. However, a single photosensitizer has limited utility to induce ER as well. Further amplification of ER stress is also essential for photosensitizers. Multifunctional nanodrugs can combine varying molecules and materials with photosensitizers into one nanoscale entity to bring the most efficient functionality against tumors.

Recently, Purushothaman et al. (2020) developed Ru-1@TPP-PEG-biotin self-assembled nanoparticles (Ru-1@TPP-PEG-biotin SAN) for cancer therapy. Ru-1@TPP-PEG-biotin SAN was loaded with ruthenium complex 1 (Ru-1, an inhibitor of chaperone GRP78 functions) as an ER stress inducer and photosensitizer. Ru-1@TPP-PEG-biotin SAN induced degradation of the lysosome and inhibition of autophagy, which benefited the release of Ru-1 and the induction of ER stress to cause strong cytotoxicity in MCF-7 and HepG2 cells. Combining photosensitizers and various ER stress inducers presented more potent and efficient antitumor effects than separately administering them. Dong et al. (2019) further developed a multifunctional photosensitizer-based nanodrug (RDDG/DC NPs) for treating breast cancer. The photosensitizers (polymers with dithiophene-benzotriazole groups, abbreviated with the letter C) and doxorubicin (a widely used chemotherapy drug, abbreviated with the letter D) were encapsulated in the ROS-sensitive dextran with 2-deoxy-glucose (2-DG, an ER stress inducer that interferes with N-linked glycosylation). The 2-DG and photosensitizers synthetically induced severe ER stress, which significantly increased CHOP mRNA (related to ER stress), and high cytotoxicity was observed in MCF-7 breast cancer cells treated with RDDG/DC NPs and light irradiation. Eventually, the well-fabricated multifunctional nanodrugs targeted the tumor site and exhibited the most potent suppressing effects on tumor growth under light illumination.

## Nanodrug-Based Strategies to Amplify Endoplasmic Reticulum Stress for Immunotherapy

Antitumor immunotherapy, which boosts the body’s own immune system’s ability to recognize and attack tumor cells, represents one of the most promising advances in modern medicine (Wang et al., 2021). However, the immunosuppressive tumor microenvironment and poor tumor immunogenicity discourage tumor cells from immune attack, which confronts immunotherapy with enormous challenges (Dong et al., 2021). ICD is a special form of cell death, activating an immune response to recognize antigens of dead or dying tumor cells (Banstola et al., 2021). The expression and release of death-association molecular patterns (DAMPs), such as CRT and high mobility group box 1 (HMGB1), promote dendritic cell (DC) maturation and antigen presentation during ICD (Kroemer et al., 2022). More immunogen exposure to tumors’ signals potentiates an immune effect, which is beneficial for treating malignant tumors. However, most ICD inducers have poor antitumor immunity because ICD-related danger signaling is not their original pharmacology mechanism but a consequence of collateral ER stress effects.

ER stress has been shown to contribute to ICD. When ER stress occurs, abundant CRTs in the ER translocate to the cell surface as a signal for immune system recognition and antigen presentation, often referred to as the “eat me” signaling. Photosensitizers have the potential to be ICD inducers due to their ability to induce ER stress, but only a small amount of them have been applied in ER-associated ICD-induced research on account of their existing but limited influences on ER stress. Some promising strategies of nanodrugs to amplify ER stress benefit antitumor immunotherapy of these limited ICD inducers.

### Endoplasmic Reticulum–Targeting Nanodrugs Based on Immunogenic Cell Death Inducers

A few ICD inducers, such as hypericin (an ER-target photosensitizer) (Lin et al., 2017), induce highly efficient ICD by directly and selectively targeting the ER. Therefore, engineering ER-targeting nanodrugs based on ICD inducers is an effective strategy for enhancing the ICD-associated antitumor immunity. Deng et al. (2020) modified meso-tetra(4-carboxyphenyl) porphyrin (TCPP) with N-tosylethylenediamine to form an ER-targeting photosensitizer TCPP-T^ER^. Notably, the *p*-toluene sulfonyl group of TCPP-T^ER^ recognized the ATP-sensitive K^+^ channel (sulfonylurea receptor) on the ER. Then, TCPP-T^ER^ was loaded to reduction-sensitive polymer (Ds-sP) to respond to the tumor microenvironment. The dual-targeting of smart Ds-sP/TCPP-T^ER^ nanoparticles released TCPP-T^ER^ in tumor sites with high GSH levels and ensured the accumulation of TCPP-T^ER^ in the ER. The ROS generated from TCPP-T^ER^ under light exposure activated ER stress directly and increased the translocation of CRTs to the cell membrane in 4T1 cells (human breast cancer cell lines). The Ds-sP/TCPP-T^ER^ nanoparticles successfully inhibited the growth of primary and distant tumors along with evaluating the proportion of CD8^+^ T cells in tumor tissues.

Additionally, Li et al. (2019) fabricated an ER target nanodrug, pardaxin (FAL) peptides–modified indocyanine green (ICG)–conjugated hollow gold nanospheres (FAL-ICG-HAuNS). The AuNS were an excellent carrier with both photothermal properties and ER stress–inducing function, which exerted a synergistic effect with photosensitizer ICG. FAL-ICG-HAuNS increased CHOP and CRTs on the cell surface under light exposure. The ER stress induced by FAL-ICG-HAuNS was ROS-dependent and was blocked by antioxidant vitamin C. To overcome the limitations of the hypoxia tumor microenvironment on PDT, the oxygen-delivering hemoglobin (Hb) liposome (FAL-Hb lipo) was adopted to provide sufficient O_2_. FAL-ICG-HAuNS inhibited tumor growth and prolonged survival time of CT-26 tumor–bearing mice and B16 tumor–bearing mice, which was reversed by depleting either CD4^+^ or CD8^+^ T cells, demonstrating the important role of ICD in tumor killing. In summary, the nanoengineered targeting strategy successfully enhanced ICD and related antitumor immunity by amplifying ER stress.

### Endoplasmic Reticulum Stress–Inducing Nanodrugs Combined With Immunogenic Cell Death Inducers

Clinically available chemotherapeutic drugs, such as DOX, platinum-based drugs, cyclophosphamide, and so on, belong to the ICD inducers *via* collateral ER stress effects (Tham et al., 2020). The increasing targeting of ER benefits the ICD-inducing ability of these inducers. However, the increasing immunogenicity is accompanied by less cytotoxicity because they initiate cell death *via* non–ER-related targets (Xiang et al., 2022b). Therefore, combining different ER stress inducers and ICD inducers to form an alternative ICD inducer with strong antitumor efficacy is a promising strategy to promote ICD-associated immunogenicity and preserve original cytotoxicity.

Recently, Xu et al. (2019) combined cisplatin and adjudin (ADD) into a multi-responsive peptide-based prodrug platform for cancer therapy. ADD was a derivative of lonidamine with potent antitumor effects, and it significantly increased intracellular ROS levels by attacking the mitochondria. The combination of cisplatin and ADD along with tumor targeting from the nanoplatform amplified CRTs exposure, ATP secretion, and HMGB-1 release. To further improve antitumor immunity, WKYMVm were loaded to nanoparticles to form 3-NPs assembled from 2-(Nap)-FFKPt-2TPA-ADDGGGPLGVRG-WKYMVm-mPEG1000. WKYMVm was an agonist of formyl peptide receptor 1 (FRR-1) which facilitated DCs to touch and contact with dying tumor cells stably. More importantly, 3-NPs demonstrated minimal lung metastases surprising rate of tumor shrinkage, as high as 93.1% in the 4T1 orthotopic tumor model. Therefore, 3-NPs were efficient in treating triple-negative breast cancer (TNBC) and inhibiting tumor metastasis *via* provoking innate and adaptive anti-TNBC immunity. In addition, Chen et al. (2019) constructed a core-shell magnetic nanoparticle (FeNP) to load oxaliplatin (the third-generation star product of platinum-based drugs) with a modified tumor-targeting peptide (α-enolase-targeting peptide, ETP) (ETP-PtFeNP). The ETP-PtFeNP induced intracellular Fenton’s reaction and elicited ROS bursting (Chen et al., 2022), which was partially beneficial for ICD. ERS-mediated ICD activation was demonstrated by markedly elevated levels of CRT on the cell surface in the ETP-PtFeNP–treated group. ETP-PtFeNP eventually activated entire-body immunity and suppressed tumor growth in the 4T1 tumor–bearing balb/c mice.

## Conclusion and Prospects

This article reviewed the tumor treatment strategies of ER stress–inducing nanodrugs. Nanodrugs are specifically enriched in tumor sites and achieve efficient treatment of cancer *via* the enhancement of ER stress. Moreover, a combination of various ER stress inducers in a nanoplatform can further amplify ER stress and demonstrate attractive antitumor effects. In addition, nanodrug-based strategies to amplify ER stress repurposed those antitumor drugs for immunotherapy and acquired amazing results, considering the importance of ER stress for ICD.

Although significant progress has been made in this emerging field, there are still some unsolved questions about these nanodrugs. First, ER exists in almost all cells, which represents the wide toxic side effects of ER-targeting nanodrugs. As such, precise tumor cell targeting is imperative. Second, many ROS-based drugs are limited by tumor environment hypoxia in tumor therapy, especially the strategy that requires direct utilization of O_2_ like PDT (Long et al., 2022). Although the work conducted by Li et al. (2019) provided a direction for breaking the restriction, there was still a long way to overcome this challenge. Finally, the ER targeting of many nanodrugs is achieved through the modification of targeting peptides, but the modification of macromolecules such as proteins increases the difficulty of synthesis. More superior ER targeting strategies, such as nanoliposome targeting ER, require further deep study (Shi et al., 2021).

Overall, nanodrugs-induced/amplified ER stress is available to increase ICD and antitumor effects. Of whatever function as cytotoxic antitumor drugs or immunotherapeutic drugs, the ER stress-inducing nanodrugs are potential and promising for cancer treatment. At the same time, the ideas of nanodrugs-based strategies based on ER stress are also beneficial for the treatment of other diseases.
